# Consumption of Meat, Fish, Dairy Products, and Eggs and Risk of Ischemic Heart Disease: A Prospective Study of 7198 Incident Cases Among 409 885 Participants in the Pan-European EPIC Cohort

**DOI:** 10.1161/CIRCULATIONAHA.118.038813

**Published:** 2019-04-22

**Authors:** Timothy J. Key, Paul N. Appleby, Kathryn E. Bradbury, Michael Sweeting, Angela Wood, Ingegerd Johansson, Tilman Kühn, Marinka Steur, Elisabete Weiderpass, Maria Wennberg, Anne Mette Lund Würtz, Antonio Agudo, Jonas Andersson, Larraitz Arriola, Heiner Boeing, Jolanda M.A. Boer, Fabrice Bonnet, Marie-Christine Boutron-Ruault, Amanda J. Cross, Ulrika Ericson, Guy Fagherazzi, Pietro Ferrari, Marc Gunter, José María Huerta, Verena Katzke, Kay-Tee Khaw, Vittorio Krogh, Carlo La Vecchia, Giuseppe Matullo, Conchi Moreno-Iribas, Androniki Naska, Lena Maria Nilsson, Anja Olsen, Kim Overvad, Domenico Palli, Salvatore Panico, Elena Molina-Portillo, J. Ramón Quirós, Guri Skeie, Ivonne Sluijs, Emily Sonestedt, Magdalena Stepien, Anne Tjønneland, Antonia Trichopoulou, Rosario Tumino, Ioanna Tzoulaki, Yvonne T. van der Schouw, W.M. Monique Verschuren, Emanuele di Angelantonio, Claudia Langenberg, Nita Forouhi, Nick Wareham, Adam Butterworth, Elio Riboli, John Danesh

**Affiliations:** 1 Nuffield Department of Population Health, University of Oxford, United Kingdom (T.J.K., P.N.A., K.E.B.).; 2 National Institute for Health Innovation, School of Population Health, University of Auckland, New Zealand (K.E.B.).; 3 Medical Research Council/British Heart Foundation Cardiovascular Epidemiology Unit, Department of Public Health and Primary Care, University of Cambridge, United Kingdom (M. Sweeting, A.W., E.d.A., A.B., J.D.).; 4 Department of Odontology, Umeå University, Sweden (I.J.).; 5 German Cancer Research Center, Division of Cancer Epidemiology, Heidelberg (T.K., V. Katzke).; 6 Medical Research Council Epidemiology Unit, University of Cambridge School of Clinical Medicine, United Kingdom (M. Steur, C.L., N.F., N.W.).; 7 Department of Community Medicine, Faculty of Health Sciences, Universitetet i Tromsø, Arctic University of Norway, Tromsø (E.W., G.S.).; 8 Department of Research, Cancer Registry of Norway, Institute of Population-Based Cancer Research, Oslo (E.W.).; 9 Department of Medical Epidemiology and Biostatistics, Karolinska Institutet, Stockholm, Sweden (E.W.).; 10 Genetic Epidemiology Group, Folkhälsan Research Center, and Faculty of Medicine, University of Helsinki, Finland (E.W.).; 11 Department of Public Health and Clinical Medicine, Nutritional Research, Umeå University, Sweden (M.W.).; 12 Section for Epidemiology, Department of Public Health, Aarhus University, Denmark (A.M.L.W., K.O.).; 13 Unit of Nutrition and Cancer, Cancer Epidemiology Research Program, Catalan Institute of Oncology–Institut d'Investigació Biomédica de Bellvitge, Barcelona, Spain (A.A.).; 14 Department of Public Health and Clinical Medicine, Research Unit Skellefteå, Umeå University, Sweden (J.A.).; 15 Public Health Division of Gipuzkoa, Instituto BIO–Donostia, Basque Government, San Sebastian, Spain (L.A.).; 16 CIBER (Biomedical Research Networking Centres) de Epidemiología y Salud Pública, Madrid, Spain (L.A., J.M.H.).; 17 Department of Epidemiology, German Institute of Human Nutrition (DIfE), Potsdam-Rehbrücke (H.B.).; 18 Centre for Nutrition, Prevention and Health Services, National Institute for Public Health and the Environment (RIVM), Bilthoven, the Netherlands (J.M.A.B.).; 19 CESP, INSERM (Centre de recherche en Epidémiologie et Santé des Populations, Institut national de la santé et de la recherche médicale) U1018, Université Paris-Sud, UVSQ, Université Paris-Saclay, Villejuif Cedex, France (F.B., M.-C.B.-R., G.F.).; 20 Gustave Roussy, Villejuif Cedex, Paris, France (F.B., M.-C.B.-R., G.F.).; 21 Department of Endocrinology, Rennes University Hospital (CHU), France (F.B.).; 22 Rennes 1 University, France (F.B.).; 23 School of Public Health, Imperial College, London, United Kingdom (A.J.C., E.R.).; 24 Department of Clinical Sciences Malmö, Lund University, Malmö, Sweden (U.E., E.S.).; 25 International Agency for Research on Cancer, World Health Organization, Lyon, France (P.F., M.G., M. Stepien).; 26 Department of Epidemiology, Murcia Regional Health Council, IMIB (Instituto Murciano de Investigación Biosanitaria)-Arrixaca, Spain (J.M.H.).; 27 Clinical Gerontology, Department of Public Health and Primary Care, School of Clinical Medicine, University of Cambridge, United Kingdom (K.-T.K.).; 28 Epidemiology and Prevention Unit, Fondazione IRCCS (Institute for Research, Hospitalization and Health Care) Istituto Nazionale dei Tumori, Milan, Italy (V. Krogh).; 29 Hellenic Health Foundation, Athens, Greece (C.L.V., A. Trichopoulou).; 30 Department of Clinical Sciences and Community Health, Università degli Studi di Milano, Milan, Italy (C.L.V.).; 31 Italian Institute for Genomic Medicine, Turin (G.M.).; 32 Department of Medical Sciences, University of Turin, Italy (G.M.).; 33 Instituto de Salud Pública de Navarra, IdiSNA–Navarre Institute for Health Research, Pamplona, Spain (C.M.-I.).; 34 World Health Organization Collaborating Center for Nutrition and Health, Unit of Nutritional Epidemiology and Nutrition in Public Health, Department of Hygiene, Epidemiology and Medical Statistics, School of Medicine, National and Kapodistrian University of Athens, Greece (A.N., A. Trichopoulou).; 35 Arctic Research Center at Umeå University, Sweden (L.M.N.).; 36 Danish Cancer Society Research Center, Copenhagen, Denmark (A.O., A.Tjønneland).; 37 Cancer Risk Factors and Life-Style Epidemiology Unit, Institute for Cancer Research, Prevention and Clinical Network–ISPRO, Florence, Italy (D.P.).; 38 Dipartimento di Medicina Clinica e Chirurgia, Federico II University, Naples, Italy (S.P.).; 39 Escuela Andaluza de Salud Pública, Instituto de Investigación Biosanitaria, Universidad de Granada, Spain (E.M.-P.).; 40 Public Health Directorate of Asturias, Oviedo, Spain (J.R.Q.).; 41 Julius Center for Health Sciences and Primary Care, University Medical Center Utrecht, the Netherlands (I.S., Y.T.v.d.S., W.M.M.V.).; 42 Cancer Registry and Histopathology Unit, “Civic-M.p.Arezzo” Hospital, ASP (Azienda Sanitaria Provinciale) Ragusa, Italy (R.T.).; 43 Department of Epidemiology and Biostatistics (I.T.), School of Public Health, Imperial College London, United Kingdom.; 44 Medical Research Council-Public Health England Centre for Environment (I.T.), School of Public Health, Imperial College London, United Kingdom.; 45 Department of Hygiene and Epidemiology, University of Ioannina Medical School, Greece (I.T.).

**Keywords:** dairy products, eggs, fish, heart diseases, meat

## Abstract

**Background:**

There is uncertainty about the relevance of animal foods to the pathogenesis of ischemic heart disease (IHD). We examined meat, fish, dairy products, and eggs and risk for IHD in the pan-European EPIC cohort (European Prospective Investigation Into Cancer and Nutrition).

**Methods:**

In this prospective study of 409 885 men and women in 9 European countries, diet was assessed with validated questionnaires and calibrated with 24-hour recalls. Lipids and blood pressure were measured in a subsample. During a mean of 12.6 years of follow-up, 7198 participants had a myocardial infarction or died of IHD. The relationships of animal foods with risk were examined with Cox regression with adjustment for other animal foods and relevant covariates.

**Results:**

The hazard ratio (HR) for IHD was 1.19 (95% CI, 1.06–1.33) for a 100-g/d increment in intake of red and processed meat, and this remained significant after exclusion of the first 4 years of follow-up (HR, 1.25 [95% CI, 1.09–1.42]). Risk was inversely associated with intakes of yogurt (HR, 0.93 [95% CI, 0.89–0.98] per 100-g/d increment), cheese (HR, 0.92 [95% CI, 0.86–0.98] per 30-g/d increment), and eggs (HR, 0.93 [95% CI, 0.88–0.99] per 20-g/d increment); the associations with yogurt and eggs were attenuated and nonsignificant after exclusion of the first 4 years of follow-up. Risk was not significantly associated with intakes of poultry, fish, or milk. In analyses modeling dietary substitutions, replacement of 100 kcal/d from red and processed meat with 100 kcal/d from fatty fish, yogurt, cheese, or eggs was associated with ≈20% lower risk of IHD. Consumption of red and processed meat was positively associated with serum non–high-density lipoprotein cholesterol concentration and systolic blood pressure, and consumption of cheese was inversely associated with serum non–high-density lipoprotein cholesterol.

**Conclusions:**

Risk for IHD was positively associated with consumption of red and processed meat and inversely associated with consumption of yogurt, cheese, and eggs, although the associations with yogurt and eggs may be influenced by reverse causation bias. It is not clear whether the associations with red and processed meat and cheese reflect causality, but they were consistent with the associations of these foods with plasma non–high-density lipoprotein cholesterol and for red and processed meat with systolic blood pressure, which could mediate such effects.

## Abstract 2

Supplemental Digital Content is available in the text.

**Clinical Perspective**

**What Is New?**

- We followed up the health of 400 000 men and women in 9 European countries for 12 years to examine the relevance of intake of animal foods to the pathogenesis of ischemic heart disease.
- Higher consumption of red and processed meat was positively associated with the risk for ischemic heart disease.
- Consumption of the other animal foods examined was not positively associated with risk; intakes of fatty fish, yogurt, cheese, and eggs were modestly inversely associated with risk.

**What Are the Clinical Implications?**

- Higher intake of red and processed meat may increase the risk of ischemic heart disease.
- Substituting other foods for red and processed meat may reduce the risk of ischemic heart disease.

Ischemic heart disease (IHD) is the commonest disease and cause of death in Europe.^1^ The risk of IHD is affected by diet, but there is uncertainty about the relevance of intake of animal foods such as red and processed meat, poultry, fish, dairy products, and eggs. Meat and dairy products are major dietary sources of saturated fatty acids; in the United Kingdom, for example, meat and meat products contribute 24% of saturated fat intake in adults, and milk and milk products contribute 22%.^2^ Controlled feeding trials have shown that high intakes of saturated fatty acids raise circulating low-density lipoprotein cholesterol, an established risk factor for IHD, suggesting that higher intakes of foods rich in saturated fatty acids may increase the risk of IHD.^3,4^ Meta-analyses of previous prospective studies of meat and the incidence of fatal IHD have suggested that intake of processed meat may be associated with higher risk, whereas intake of unprocessed red meat might not.^5,6^ For dairy products and eggs, systematic reviews of prospective studies have reported no consistent evidence that higher intakes are associated with a higher risk of IHD.^7,8^ Consumption of fatty fish might reduce the risk of IHD because it is a rich source of long-chain n-3 fatty acids, and a meta-analysis has suggested an inverse association between overall fish consumption and mortality from IHD.^9^

Here, we report the relationships of these foods with the risk of IHD in EPIC (European Prospective Investigation Into Cancer and Nutrition), a cohort of half a million men and women.^10,11^ To assess whether associations might be the result of reverse causation, we examined the results after excluding the first 4 years of follow-up. To assess whether associations might be explained by known metabolic risk factors for IHD, we examined the cross-sectional associations of food intake with cholesterol fractions and blood pressure in a subsample of participants, interpreting the relationships of foods with risk with respect to their associations with non–high-density lipoprotein (HDL) cholesterol and systolic blood pressure.

## Methods

Because of the sensitive nature of the data collected for this study, requests to access the data set from qualified researchers trained in human subject confidentiality protocols may be sent to the International Agency for Research on Cancer at http://epic.iarc.fr/access/index.php.

### Study Population

EPIC is a prospective study of ≈520 000 men and women recruited through 23 centers in 10 European countries, mostly between 1992 and 2000.^10,11^ Participants in EPIC completed dietary and lifestyle questionnaires, and the majority also provided blood samples and had their blood pressure measured. The baseline data were centralized at the World Health Organization’s International Agency for Research on Cancer in Lyon, France. All participants gave written informed consent, and the study protocol was approved by the ethics review boards of the International Agency for Research on Cancer and the institutions where participants were recruited.^10^

Dietary intake during the year before enrollment was measured by country-specific diet assessment methods, in most centers food frequency questionnaires; these were validated with a standardized, coordinated approach.^10^ Dietary intakes estimated with a standardized and computerized 24-hour recall method were also collected from an 8% random sample across all centers ≈1.4 years after recruitment. The sample was stratified by age and sex, with weighting according to predicted disease rates in these strata, and distributed equally by season and day of the week.^12^ Details of the categorization of foods are given in the online-only Data Supplement.

Assessments of the nondietary variables were based on responses in the baseline questionnaires and categorized into the following groups: smoking (never, former, current <10 or unknown number of cigarettes per day, current 10–19 cigarettes per day, current ≥20 cigarettes per day, or unknown [2.4% of the cohort]); alcohol intake (not current drinker, sex-specific fifths of current intake: cut points in men were 3.5, 9.7, 18.8, and 36.2 g/d; cut points in women were 0.9, 2.8, 6.9, and 13.9 g/d); physical activity (Cambridge physical activity index, based on occupational physical activity and cycling/other physical exercise and categorized in approximate quartiles called inactive, moderately inactive, moderately active, active, and unknown [2.2%])^13^; highest education level obtained (none or primary school only, secondary school, vocational qualification or university degree, unknown [4.3%]); employment status (currently employed or student, neither, unknown [11.4%]); and history of diabetes mellitus, hypertension, and hyperlipidemia (each self-reported: yes, no, unknown [4.2%, 5.5%, and 23.7%, respectively]). Body mass index (BMI; <22.5, 22.5–24.9, 25.0–27.4, 27.5–29.9, ≥30.0 kg/m^2^, and unknown [0.9%]) was calculated from measured height and weight (except for participants in Norway and some participants in France and the United Kingdom, for whom height and weight were self-reported). Baseline systolic and diastolic blood pressures were measured in millimeters of mercury by trained personnel (further details are given in the online-only Data Supplement).^14^

Lipids were measured in stored plasma samples as part of the EPIC-CVD (Cardiovascular Disease) case-cohort study, which is nested within EPIC.^11^ The subcohort was randomly selected from participants with a stored blood sample, with selection stratified by the 23 EPIC recruitment centers. Details of methods are given in the online-only Data Supplement.

### Ascertainment and Verification of Cases of IHD

The outcome was IHD, defined as the composite of first nonfatal myocardial infarction (MI; *International Classification of Diseases, 10th Revision* code I21) or death resulting from IHD (*International Classification of Diseases, 10th Revision* codes I20–I25). Incident nonfatal MIs were ascertained in each EPIC center with a combination of record linkage to morbidity or hospital registries and self-reports followed by confirmation with medical records.^11^ Information on vital status was collected from mortality registries at the regional or national level in most centers except in Greece, where vital status was ascertained by active follow-up of study participants and next of kin. Centers in Denmark, Greece, Italy, Norway, and Spain validated all suspected cases of MI, whereas centers in France, the Netherlands, Sweden, and the United Kingdom validated a subset of the suspected cases to assess the accuracy of the overall ascertainment process. A range of methods was used to confirm the diagnosis of IHD and included retrieving and assessing medical records or hospital discharge notes, contact with medical professionals, retrieval and assessment of death certificates, or verbal autopsy with the next of kin. The last year of follow-up varied across centers between 2003 and 2010 but was mainly 2008 or 2009.

### Statistical Analysis

Of the 518 502 participants for whom data were available, those with no dietary data, no nondietary (lifestyle) data, and those in the top or bottom 1% of the ratio of energy intake to energy requirement were excluded (n=16 837), as were those who had a self-reported or unknown history of MI or stroke at baseline (n=11 308), 23 cases whose date of diagnosis was after the end of follow-up for each center, and 23 participants with no follow-up data. These exclusions left a total of 490 311 participants, and further restricting the data set to EPIC centers with known values for all of the animal foods (which meant excluding Heidelberg, Potsdam, Naples, and Umeå) left a total of 409 885 participants, including 7198 incident cases of nonfatal MI (n=5392) or fatal IHD (n=1806).

Follow-up was measured from recruitment until the date of first nonfatal MI or fatal IHD event or censoring at the date of death from other causes, nonfatal non-MI IHD, the date at which follow-up for IHD events was considered complete, or emigration or other loss to follow-up (1.3%). Relative risks as hazard ratios (HRs) and their 95% 95% CIs were estimated with Cox regression models. All analyses were stratified by sex and EPIC center and adjusted for exact age at recruitment (continuous); smoking; self-reported history of diabetes mellitus, hypertension, and hyperlipidemia; physical activity; employment status; level of education; BMI (these last 8 covariates were all categorical variables, with unknown categories added); current alcohol consumption (categorical); and intakes of energy, fruit and vegetables, dietary fiber from cereals, and percent energy from sugars (each continuous). In the main analyses of calibrated food intakes, the results for each animal source food were also adjusted simultaneously for the other animal source foods.

Participants were divided into fifths of self-reported intake for each animal food according to the recruitment questionnaire (for any foods with >20% zero values, the categories were approximate fifths), with the quintiles calculated for all included participants, and a trend test was performed by scoring the categorical fifths of intake 1 to 5 and treating this as a continuous variable. To test for whether the data were compatible with a linear trend, we also fitted models with the fifths of intake treated as a categorical variable. There were no significant improvements in fit when we compared the categorical intake model with the continuous (trend test) intake model, suggesting that any associations between food intake and risk were approximately linear. Then, to improve the comparability of dietary data across participating centers and to correct for measurement error in relative risk estimates, the dietary data from the subset of participants with 24-hour recalls were used to provide statistically calibrated estimates of dietary intakes for all included participants. HRs were calculated for increments in observed and calibrated intake of each food. Observed food intakes were calibrated with a fixed-effect linear model in which center- and sex-specific 24-hour recall data from an 8% random sample of the cohort were regressed on the observed intakes, generating a calibrated intake corresponding to each observed intake.^12,15^ The sizes of the increments were chosen to approximate the difference in mean 24-hour recall intake between participants in the lowest and highest fifths of observed intake and with reference to the increments used in previous publications such the World Health Organization’s review of the carcinogenicity of red and processed meat.^16^

Using the results from the mutually adjusted risks model, we estimated the effects of substituting 100 kcal/d of each other animal food for 100 kcal/d of red and processed meat from the ratios of the risk (as measured by the HR) for each food in turn and the risk for red and processed meat.^17^ For example, if P and R represent the HRs per 100 kcal/d yogurt and per 100 kcal/d red and processed meat in the mutually adjusted risks model, the effect of substituting 100 kcal/d yogurt for 100 kcal/d red and processed meat is estimated by the ratio P/R; the difference in covariance was used to estimate the 95% CI.

To examine whether the overall results might be influenced by reverse causality, we repeated the analyses after excluding the first 4 years of follow-up (ie, with follow-up for all participants beginning 4 years after the date of recruitment). To examine whether associations between the animal foods and IHD risk were consistent across subgroups of other risk factors, we also conducted separate analyses for subsets of sex, smoking status (never, former, and current), prior disease status (participants with or without a history of diabetes mellitus, hypertension, or hyperlipidemia), age at recruitment (<55, 55–64, ≥65 years), BMI (<25.0, 25.0–29.9, ≥30.0 kg/m^2^), European region (Northern Europe: Denmark, Norway, and Sweden; Central Europe: France excepting Provence and Southwest France, the Netherlands, and the United Kingdom; Southern Europe: Greece, Italy, Spain, Provence, and Southwest France), and countries with partial (France, Netherlands, Sweden, and the United Kingdom) or complete (Denmark, Greece, Italy, Norway, and Spain) validation of cases. Tests for heterogeneity of trend between subgroups were obtained by comparing the risk coefficients for each subgroup using inverse variance weighting, testing for statistical significance with a χ^2^ test on k−1 *df*, where k is the number of subgroups.

To examine whether dietary risk factors might act through major established physiological IHD risk factors, we examined the associations of food intakes with non-HDL cholesterol and systolic blood pressure, calculating mean levels of these biomarkers in each category of animal food intake (using linear regression to estimate least-squares means), with adjustment for age, sex, and EPIC center.

All analyses were performed with Stata version 15.1 (Stata Corp, College Station, TX), and a value of *P*<0.05 was considered statistically significant.

## Results

After a mean follow-up of 12.6 years, there were 7198 incident cases of MI or death resulting from IHD. Table 1 shows participant characteristics by sex for all cohort participants and for incident cases. On average, cases were 6 to 10 years older than average for the cohort, with higher mean BMI and lower mean alcohol intake. Cases were more likely to smoke; to be inactive, unemployed, and diabetic; and to have elevated blood pressure or proatherogenic lipids, lower mean observed intakes of fruit and vegetables; there were moderate differences in intakes of animal foods.

**Table 1.**
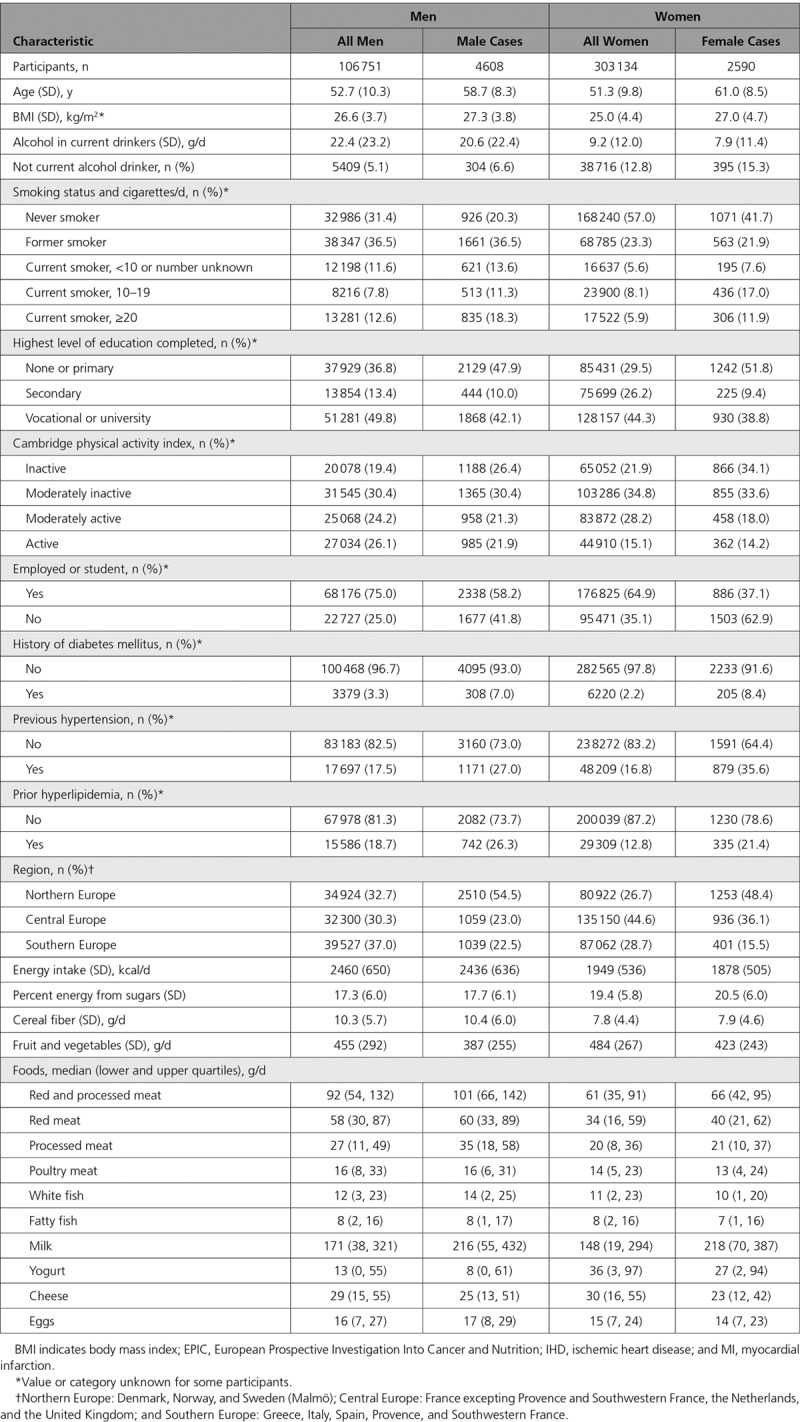
Participant Characteristics at Recruitment in 409 885 Participants by Sex and Incident Case Status for First Nonfatal MI or Fatal IHD: EPIC Study

Table 2 shows the HRs and 95% CIs for IHD in each fifth of observed intake of animal foods, relative to the bottom fifth of intake, and *P* values for tests of trend based on the observed intakes. HRs in the top fifth of intake compared with the bottom fifth of intake were 1.13 (95% CI, 1.02–1.26) for red and processed meat combined, 1.10 (95% CI, 0.99–1.21) for red meat, and 1.10 (95% CI, 0.99–1.22) for processed meat. Intakes of poultry, white fish, fatty fish, milk, and eggs were not associated with IHD, whereas intakes of yogurt and cheese were inversely associated with risk, with HRs in the top fifths of 0.90 (95% CI, 0.84–0.97) and 0.88 (95% CI, 0.80–0.96)

**Table 2.**
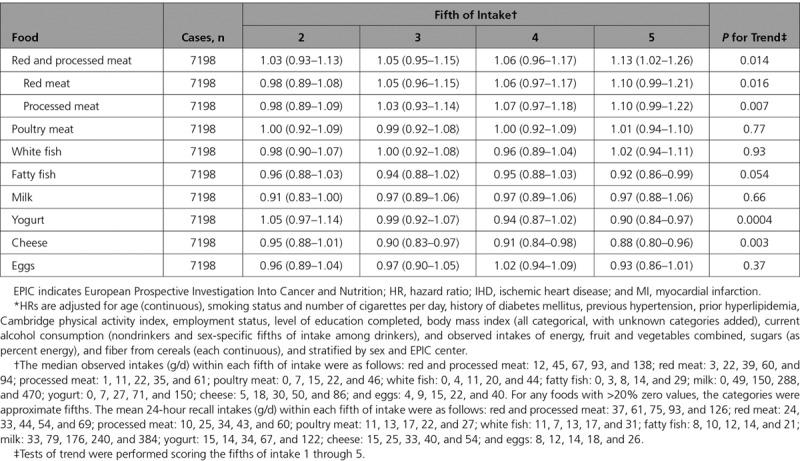
HRs* (95% CIs) for First Nonfatal MI or Fatal IHD in 409 885 Participants by Overall Fifths of Observed (Self-Reported) Intake of Selected Animal Foods, Relative to the Bottom Fifth of Intake: EPIC Study

The Figure shows the associations of IHD risk with statistically calibrated increments in intake of 8 mutually exclusive animal foods (including red and processed meat combined but not red meat and processed meat separately), with mutual adjustment of risks for the animal foods (Table I in the online-only Data Supplement gives HRs for uncalibrated and calibrated increments without mutual adjustment). For red and processed meat combined, the HR was 1.19 (95% CI, 1.06–1.33) for a 100-g/d increment in calibrated intake. The HRs for calibrated intakes of yogurt (100 g/d), cheese (30 g/d), and eggs (20 g/d) were 0.93 (95% CI, 0.89–0.98), 0.92 (95% CI, 0.86–0.98), and 0.93 (95% CI, 0.88–0.99), respectively.

**Figure.**
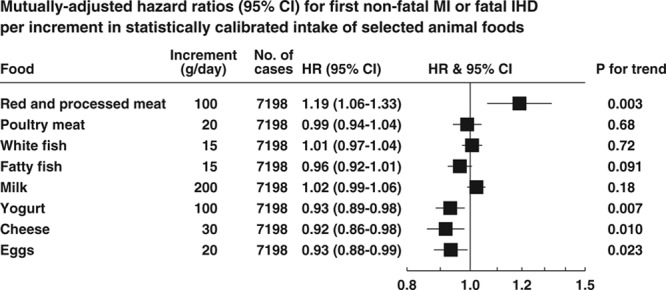
**Mutually adjusted hazard ratios (HRs; 95% CIs) for first nonfatal myocardial infarction or fatal ischemic heart disease per increment in statistically calibrated intake of animal foods.** HRs are adjusted for age (continuous), smoking status and number of cigarettes per day, history of diabetes mellitus, previous hypertension, prior hyperlipidemia, Cambridge physical activity index, employment status, level of education completed, body mass (all categorical, with unknown categories added), current alcohol consumption (nondrinkers and sex-specific fifths of intake among drinkers), and calibrated intakes of energy, fruit, and vegetables combined, sugars (as percent energy), fiber from cereals, and each other food (each continuous), and stratified in the analysis by sex and EPIC (European Prospective Investigation Into Cancer and Nutrition) center. HR indicates hazard ratio; IHD, ischemic heart disease; and MI, myocardial infarction.

In analyses excluding the first 4 years of follow-up, the association of risk with intake of red and processed meat was marginally stronger (HR per 100-g/d increment 1.25 [95% CI, 1.09–1.42]; *P*=0.001), whereas the associations with calibrated intakes of yogurt and eggs were attenuated, and neither these associations nor the association with cheese was statistically significant (Table 3).

**Table 3.**
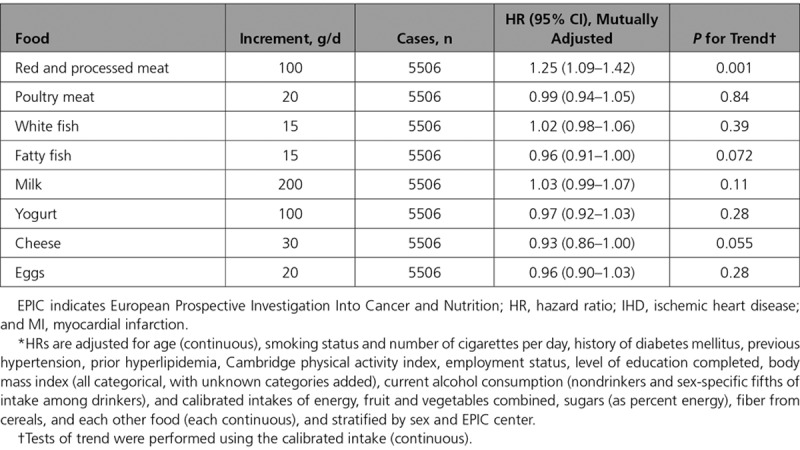
Mutually adjusted HRs* (95% CIs) for First Nonfatal MI or Fatal IHD in 406 908 Participants per Increment in Calibrated Intake of Selected Animal Foods After Exclusion of the First 4 Years of Follow-Up: EPIC Study

### Substitution Analyses

Table 4 shows the HRs for modeled substitution of 100 kcal/d of calibrated intake of red and processed meat by 100 kcal/d of each of the other animal foods. Fatty fish, yogurt, cheese, and eggs were associated with significantly lower risks for IHD than red and processed meat (15%–24% reductions in risk per 100 kcal substituted per day).

**Table 4.**
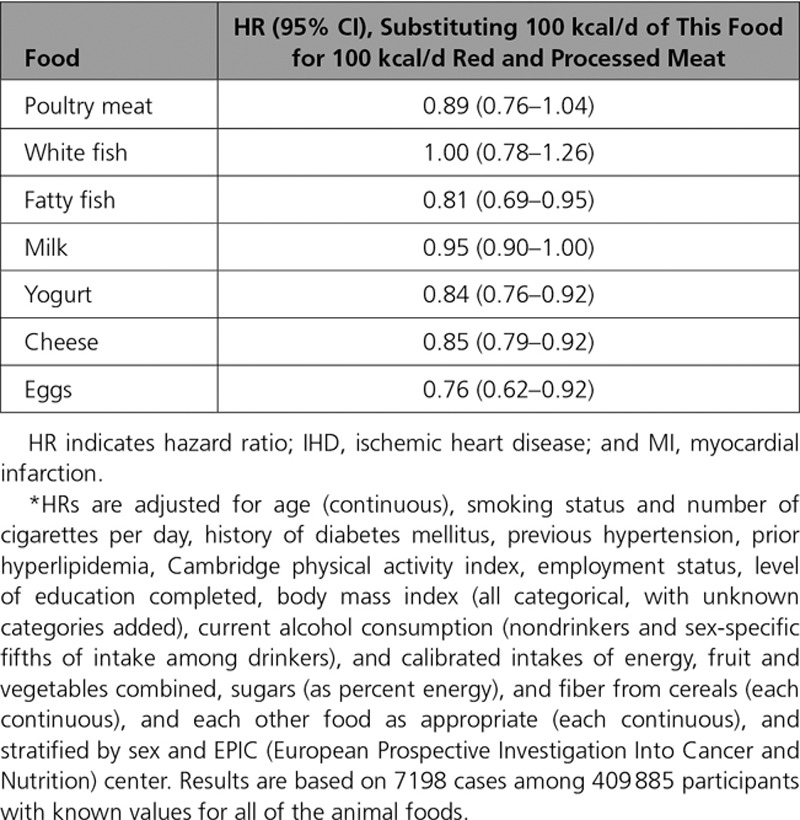
HRs* (95% CIs) for First Nonfatal MI or Fatal IHD for Substitution of 100-kcal/d Increment in Calibrated Energy Intake From Each Food for 100-kcal/d Increment in Calibrated Energy Intake From Red and Processed Meat

### Subgroup Analyses

In analyses subdivided by history of diabetes mellitus, previous hypertension, or hyperlipidemia, there was no appreciable heterogeneity in the associations of animal foods with IHD risk except for white fish, but this was not significantly associated with risk in either subgroup (see Methods and Table II in the online-only Data Supplement). In analyses subdivided by smoking status, there was no appreciable heterogeneity in the associations of animal foods with IHD risk except for yogurt, which was inversely associated with risk in current smokers but not in never smokers or former smokers (Table III in the online-only Data Supplement). In analyses subdivided by age, there was no appreciable heterogeneity in the associations of animal foods with IHD risk except for red and processed meat, which was strongly positively associated with risk in participants recruited at <55 years of age but not in older people (Table IV in the online-only Data Supplement). In analyses subdivided by sex, there was no appreciable heterogeneity in the associations of animal foods with IHD risk except for eggs, which were inversely associated with risk in men but not in women (Table V in the online-only Data Supplement). There was no appreciable heterogeneity in the associations of animal foods with IHD risk subdivided by BMI or by European region (Tables VI and VII in the online-only Data Supplement). There was evidence of heterogeneity by the extent of validation of cases in the associations of dietary intake with IHD risk for red and processed meat and for milk (Table VIII in the online-only Data Supplement); for red and processed meat, there was a large and highly significant association with risk in the countries with complete case verification but not in the other countries. For milk, there was a small positive association with risk in the countries with complete verification but not in the other countries.

### Associations of Foods With Plasma Lipids and Blood Pressure

In a comparison of the participants in the highest fifth of intake of red and processed meat with those in the lowest fifth of such intake, non-HDL cholesterol was higher by 0.19 mmol/L (4.3%), and systolic blood pressure was higher by 3.3 mm Hg (2.5%). For processed meat, the difference in systolic blood pressure between these groups of participants was 3.7 mm Hg (2.8%). Comparing participants in the highest fifth of intake of cheese with those in the lowest fifth of such intake showed that non-HDL cholesterol was lower by 0.10 mmol/L, whereas the intake of cheese was unrelated to systolic blood pressure (see Tables IX and X in the online-only Data Supplement).

## Discussion

In this large European cohort, we observed a positive association between red and processed meat intake and risk of IHD, with a 19% (95% CI, 6–33) higher risk per 100-g/d increment in calibrated intake. Red and processed meat showed separate (albeit borderline significant) associations with risk, which were each of similar magnitude. The association of risk with red and processed meat was observed after exclusion of the first 4 years of follow-up and in participants without diabetes mellitus, elevated blood pressure, or proatherogenic lipids. These additional results therefore reduce the likelihood of reverse causation or residual confounding. In comparison, a previous meta-analysis of meat intake and risk of IHD reported that unprocessed red meat consumption was not associated with risk of IHD, whereas processed meat was, with a 42% higher risk per 50-g/d increment in intake.^5^ However, that previous review included only 769 events from 4 studies for unprocessed red meat, including 1 case-control study; for processed meat, it included 21 308 events from 5 studies, but most cases derived from 1 study for which the end point was total cardiovascular mortality rather than incident MI and fatal IHD. A subsequent meta-analysis also concluded that processed meat but not unprocessed red meat was associated with IHD mortality, on the basis of up to 1370 deaths resulting from IHD.^6^ Hence, further work is needed to understand potential reasons for the differences in the results of the current study, which were based on >7000 IHD events.

We observed no clear association of IHD risk with consumption of either white fish or fatty fish (although there were a borderline significant inverse association for fatty fish and a significant inverse association for fatty fish in the substitution analyses; see below). The possible protective role of fish in IHD has been investigated for >30 years. A previous analysis of fish consumption and mortality in EPIC found no evidence that higher intakes of total, white, or fatty fish were associated with mortality from IHD.^18^ In contrast, a meta-analysis of 4472 deaths in 17 cohort studies suggested that there was an overall significant inverse association between fish intake and IHD mortality, but the association was not linear, and the relative risk in the highest category of fish intake was not significantly lower than that in the lowest intake.^9^

Dairy products are a major source of dietary saturated fatty acids, but prospective observational studies have generally not shown a higher risk of IHD with a higher intake of foods such as milk, yogurt, and cheese.^19,20^ We observed no association of milk with risk of IHD, which is consistent with a meta-analysis of 4391 incident IHD cases in 6 prospective studies.^21^ We observed that yogurt consumption was inversely associated with risk of IHD. However, this association did not persist after exclusion of the initial 4 years of follow-up, and it showed heterogeneity by smoking status, with no association in never smokers (suggesting therefore that the observed association may partly be explained by changes in diet resulting from preclinical disease or residual confounding by smoking). Yogurt consumption is associated with healthy dietary patterns, behaviors, and lifestyle factors,^22^ yet a meta-analysis of 5 prospective studies (number of cases unclear) reported no association between yogurt consumption and risk of IHD.^23^ We also observed that cheese consumption was inversely associated with risk of IHD; again, this inverse association was not significant after we excluded the first 5 years of follow-up, although the estimate was only slightly attenuated. A meta-analysis of 8 prospective studies with 7425 incident cases showed a lower risk for IHD in participants with a relatively high intake of cheese.^24^ It has been suggested that cheese has constituents that might act to reduce the risk of IHD, for example, that the calcium in cheese forms insoluble soaps with fatty acids, thus reducing absorption of saturated fatty acids, and that the calcium also binds to bile acids, reducing their enterohepatic circulation and possibly leading to a cholesterol-lowering effect.^19,25^

Egg consumption was inversely associated with IHD risk overall, but this association was no longer evident after exclusion of the first 4 years of follow-up, perhaps because of limited power or because people with preclinical disease may have reduced their egg consumption. A recent meta-analysis of 6 prospective studies including 5847 incident cases reported no association of egg consumption with risk of coronary heart disease,^8^ whereas a recent large prospective study in China including 31 169 incident cases of IHD reported that egg intake was inversely associated with risk^26^; it is possible that the risk associations found in the observational studies resulted from the dietary pattern often accompanying high egg intake or the cluster of other risk factors in people with high egg consumption.^27^

The positive association we observed between red and processed meat and risk of IHD might be related to the saturated fat content of these foods. However, although dairy products are also relatively rich in saturated fats, intake of dairy products was not positively related to IHD risk in this study; in fact there was a suggestion of an inverse association between cheese intake and future risk of IHD. This finding might suggest that different food sources of saturated fat and different proportions of individual saturated fatty acids contained within meat and dairy foods may differ in their impact on risk of IHD, which would affect the interpretation of previous studies of total dietary saturated fatty acids and risk.^28^ It is also possible that plant sources of protein may be associated with a lower risk of IHD than animal foods,^29^ and this should be considered in future analyses.

### Substitution of Other Animal Foods for Red and Processed Meat

Our analyses showed that red meat and processed meat were positively associated with risk for IHD, whereas the other animal foods were not associated or were inversely associated with risk. We therefore conducted analyses modeling isocaloric dietary substitutions, which showed that fatty fish, yogurt, cheese, and eggs were associated with significantly lower risks for IHD when substituted for red and processed meat (15%–24% lower risk per 100 kcal substituted per day). Plant foods might also be associated with a lower risk of cardiovascular disease than animal foods^27^ and may be considered in future analyses.

### Possible Roles of Plasma Lipids and Blood Pressure

The positive associations of red and processed meat and the inverse association of cheese consumption with the risk of IHD might be explained by the associations of these foods with well-established risk factors for IHD such as cholesterol fractions and systolic blood pressure. Compared with participants in the lowest fifth of intake of red and processed meat, those in the top fifth had a higher non-HDL cholesterol by 0.19 mmol/L and a higher systolic blood pressure by 3.3 mm Hg; the difference in systolic blood pressure was larger for processed meat than for red meat (3.7 and 2.2 mm Hg, respectively), consistent with previous observations and possibly caused by the high salt content of most processed meats.^30^

On the basis of results from the Emerging Risk Factors Collaboration and the Prospective Studies Collaboration,^31,32^ these differences would be expected to be associated with higher IHD risks of 8% and 12%, respectively. Such modeling suggests that the observed (uncalibrated) 13% higher risk in the top fifth of intake of red and processed meat could be readily explained by the differences in blood lipids and blood pressure. Other mechanisms might also be involved; for example, higher intakes of red and processed meat might increase the risk of IHD through the conversion of carnitine in meat into trimethylamine oxide.^33^ Compared with participants in the lowest fifth of intake of cheese, those in the top fifth had lower non-HDL cholesterol by 0.10 mmol/L but no significant difference in systolic blood pressure. Again on the basis of results from the Prospective Studies Collaboration, this difference in lipids would be expected to be associated with a 4% lower IHD risk, indicating that the observed 12% lower IHD risk in the top fifth of intake of cheese might only partly be explained by standard lipid fractions.

### Strengths and Limitations

Strengths of this study are the large number of cases, the prospective design, the wide range of diets across Europe, the calibration of the dietary data with 24-hour recalls, and the ability to adjust for major risk factors for IHD and to estimate the impacts of associations with circulating lipids and blood pressure.

As with all observational studies, a potential limitation is that the associations may be influenced by confounding by other risk factors. We have adjusted our results for major risk factors for IHD, including smoking and BMI, as well as socioeconomic factors. However, because the magnitudes of the associations we observed were relatively modest, we cannot discount that the results have been influenced by residual confounding by adiposity, socioeconomic factors, or other unmeasured factors. Another potential limitation is that, because of the multicenter design of the cohort, there were some variations in the ascertainment and validation of the end point; the positive association of red and processed meat with risk for IHD was strong in the countries with complete validation of cases. It is also possible that associations of specific foods with risk may vary between populations as a result of differences in associations with other aspects of diet.

## Conclusions

This large prospective study in Europe shows a moderate positive association between consumption of red and processed meat and risk of IHD, and it suggests a modest inverse association between consumption of cheese and IHD risk. It is not clear whether these associations reflect causality, but they were consistent with the associations of these foods with plasma non-HDL cholesterol and for red and processed meat with systolic blood pressure, which could mediate such effects.

## Acknowledgments

The authors thank all EPIC participants and staff for their contributions to the study. They thank staff from the EPIC-CVD and EPIC-InterAct Coordinating Centres for carrying out sample preparation and data-handling work, particularly Sarah Spackman (EPIC-CVD data manager) and Nicola Kerrison (EPIC-InterAct data manager). They thank the EPIC-InterAct project (http://www.interact.eu/) for use of the data on plasma lipids. The author acknowledge Statistics Netherlands for providing information on causes of death to the Dutch EPIC Centres. The study was conceived and designed by T.J.K., P.N.A., K.E.B., A.B., E.R., and J.D. The data were analyzed by P.N.A. The first draft of the manuscript was prepared by T.J.K., P.N.A., and K.E.B. and edited with input from the writing team (I.J., T.K., M.S., E.W., M.W., and A.M.L.W.). All other authors provided the data and revised the manuscript critically for important intellectual content. All authors gave final approval of the version to be published and have contributed to the manuscript. T.J.K. is the guarantor.

## Sources of Funding

Analyses were supported by the UK Medical Research Council (MR/M012190/1), Cancer Research UK (C8221/A19170 and 570/A16491), and the Wellcome Trust (Our Planet Our Health, Livestock Environment and People 205212/Z/16/Z). The project (EPIC-CVD) has been supported by the European Union Framework 7 (HEALTH-F2-2012–279233), the European Research Council (268834), the UK Medical Research Council (G0800270 and MR/L003120/1), the British Heart Foundation (SP/09/002, RG/08/014, and RG13/13/30194), and the UK National Institute of Health Research. The coordination of EPIC is financially supported by the European Commission and the International Agency for Research on Cancer. The national cohorts are supported by Danish Cancer Society (Denmark); Ligue Contre le Cancer, Institut Gustave Roussy, Mutuelle Générale de l’Education Nationale, and Institut National de la Santé et de la Recherche Médicale (INSERM) (France); German Cancer Aid, German Cancer Research Center, Federal Ministry of Education and Research, Deutsche Krebshilfe, Deutsches Krebsforschungszentrum, and Federal Ministry of Education and Research (Germany); the Hellenic Health Foundation (Greece); Italian Association for Research on Cancer, National Research Council (Italy) and Ministero dell’Istruzione dell’Università e della Ricerca “Dipartimenti di Eccellenza” (Project D15D18000410001) to the Department of Medical Sciences (Italy); Dutch Ministry of Public Health, Welfare and Sports, Netherlands Cancer Registry, LK Research Funds, Dutch Prevention Funds, Dutch Zorg Onderzoek Nederland, World Cancer Research Fund; Health Research Fund, PI13/00061 to Granada, PI13/01162 to EPIC-Murcia, regional governments of Andalucía, Asturias, Basque Country, Murcia (No. 6236), and Navarra, Instituto de Salud Carlos III (ISCIII RETIC RD06/0020) (Spain); Swedish Cancer Society, Swedish Research Council and County Councils of Skåne and Västerbotten (Sweden); Cancer Research UK (14136 to EPIC-Norfolk; C570/A16491 and C8221/A19170 to EPIC-Oxford), UK Medical Research Council (1000143 to EPIC-Norfolk, MR/M012190/1 to EPIC-Oxford, MC_UU_12015/1 (Drs Langenberg and Wareham), and MC_UU_12015/5 (Dr Forouhi), and National Institute for Health Research Biomedical Research Center Cambridge: Nutrition, Diet, and Lifestyle Research Theme (IS-BRC-1215–20014) to the Medical Research Council Epidemiology Unit Cambridge. Dr Bradbury holds the Girdlers’ New Zealand Health Research Council Fellowship. Dr Steur received Core Medical Research Council Unit support through the Nutritional Epidemiology Program (MC_UU_12015/5) while at the Medical Research Council Epidemiology Unit and received funding from the Alpro Foundation while at the Cardiovascular Epidemiology Unit. John Danesh holds a British Heart Foundation Professorship, National Institute for Health Research Senior Investigator Award, and European Research Council Senior Investigator Award. The authors assume full responsibility for analyses and interpretation of these data.

## Disclosures

None.

## Supplementary Material
