# Supplementary material for: Consumption of Meat, Fish, Dairy Products, and Eggs and Risk of Ischemic Heart Disease: A Prospective Study of 7198 Incident Cases Among 409 885 Participants in the Pan-European EPIC Cohort
Source: Circulation. 2019 Apr 22;139(25):2835–45. doi: 10.1161/CIRCULATIONAHA.118.038813 (PMC6629175; doi:10.1161/CIRCULATIONAHA.118.038813)
Supplement: Supplementary file 1 [file cir-139-2835-s001.pdf]

SUPPLEMENTAL MATERIAL

Meat, fish, dairy products, eggs and risk of ischaemic heart disease: a prospective study of 7198 incident cases among 409885 participants in the pan-European EPIC cohort

Supplemental Methods

Meats were categorized as red meats (unprocessed, including beef, veal, pork, lamb, mutton, goat, horse, hamburger, meatballs, minced meat), processed meat (meat products, including poultry, preserved by methods other than freezing, such as salting with or without nitrites, smoking, marinating, air drying, or heating, and including ham, bacon, sausages, blood sausages, chicken sausage, meat cuts, liver paté, salami, bologna, tinned meat, luncheon meat, corned beef and black pudding), and poultry (excluding processed poultry). Fish was categorized as white fish and fatty fish. Dairy products were categorized as milk (plain milk including buttermilk, excluding milk-based beverages), yogurt and cheese, and eggs were treated as a single item. Data on white fish were not available for the centres in Naples, Heidelberg and Potsdam, data on fatty fish were not available for the centre in Potsdam, and data on eggs were not available for the centre in Umeå, so data from these centres were excluded.

Total cholesterol was measured in all cases with stored blood samples and the subcohort by enzymatic assay at Stichting Huisartsen Laboratorium (Etten-Leur, Netherlands), and an enzymatic colorimetric assay method (Roche) was used to measure HDL cholesterol directly. Lipid measurements were not available for participants from Norway. Blood pressure was measured at the time of recruitment for most participants. Systolic and diastolic blood pressures were measured by trained personnel; two readings were performed on the right arm in a sitting position (spaced by 1–5 minutes) after an initial resting time of at least 5 minutes by use of a standard mercury manometer or oscillometric device (with the exception of the Danish and Swedish centres where one single measurement was taken in the supine position). Blood pressure was not available for participants from France, Norway, and Asturias and Navarra in Spain.

Supplemental Tables

Supplemental Table 1 Hazard ratios* (95% confidence intervals) for first non-fatal MI or fatal IHD in 409885 participants per increment in observed (self-reported) and statistically calibrated intake of selected animal foods

Food	Increment (g/day)	No. of cases	Observed intake	P for trend#	Calibrated intake	P for trend#
Red and processed meat	100	7198	1.12 (1.05-1.18)	0.0002	1.22 (1.09-1.37)	0.0004
Red meat	50	7198	1.06 (1.02-1.10)	0.002	1.10 (1.02-1.19)	0.018
Processed meat	50	7198	1.05 (1.00-1.10)	0.035	1.14 (1.04-1.24)	0.003
Poultry meat	20	7198	1.00 (0.98-1.03)	0.86	0.99 (0.94-1.04)	0.74
White fish	15	7198	1.00 (0.98-1.02)	0.82	1.00 (0.97-1.04)	0.92
Fatty fish	15	7198	0.98 (0.96-1.01)	0.15	0.96 (0.92-1.00)	0.063
Milk	200	7198	1.01 (0.99-1.03)	0.36	1.02 (0.99-1.06)	0.17
Yogurt	100	7198	0.95 (0.93-0.98)	0.002	0.92 (0.88-0.97)	0.002
Cheese	30	7198	0.97 (0.94-0.99)	0.014	0.90 (0.85-0.97)	0.003
Eggs	20	7198	0.97 (0.94-1.00)	0.026	0.94 (0.88-1.00)	0.034

* Hazard ratios are adjusted for age (continuous), smoking status and number of cigarettes per day, history of diabetes, previous hypertension, prior hyperlipidemia, Cambridge physical activity index, employment status, level of education completed, BMI (all categorical, with 'unknown' categories added), current alcohol consumption (non-drinkers and sex-specific fifths of intake among drinkers), and observed/calibrated intakes of energy, fruit and vegetables combined, sugars (as % energy) and fibre from cereals (each continuous), and stratified by sex and EPIC centre.

# Tests of trend were performed using the observed or calibrated intake (both continuous).

Supplemental Table 2 Mutually-adjusted hazard ratios* (95% confidence intervals) for first non-fatal MI or fatal IHD per increment in calibrated intake of selected animal foods, subdivided by prior disease status in 322592 participants with known prior disease status

Food	Increment (g/day)	Participants with **no** history of diabetes, hypertension or hyperlipidemia			Participants with a history of diabetes, hypertension or hyperlipidemia			P for heterogeneity[&]
		No. of cases	HR (95% CI)	P for trend[#]	No. of cases	HR (95% CI)	P for trend[#]	
Red and processed meat	100	2293	1.23 (1.01-1.50)	0.039	2895	1.09 (0.91-1.31)	0.35	0.38
Poultry meat	20	2293	0.99 (0.91-1.07)	0.78	2895	1.01 (0.95-1.08)	0.71	0.65
White fish	15	2293	1.06 (0.99-1.14)	0.072	2895	0.97 (0.92-1.02)	0.20	0.027
Fatty fish	15	2293	1.01 (0.93-1.09)	0.83	2895	0.96 (0.90-1.02)	0.19	0.32
Milk	200	2293	1.00 (0.94-1.05)	0.89	2895	1.03 (0.97-1.08)	0.32	0.44
Yogurt	100	2293	0.88 (0.79-0.98)	0.016	2895	0.93 (0.85-1.01)	0.082	0.44
Cheese	30	2293	0.91 (0.81-1.02)	0.12	2895	1.00 (0.90-1.11)	0.95	0.26
Eggs	20	2293	0.95 (0.86-1.05)	0.30	2895	0.92 (0.84-1.02)	0.10	0.72

* Hazard ratios are adjusted for age (continuous), smoking status and number of cigarettes per day, histories of diabetes, hypertension and hyperlipidemia (where appropriate), Cambridge physical activity index, employment status, level of education completed, BMI (all categorical, with 'unknown' categories added), current alcohol consumption (non-drinkers and sex-specific fifths of intake among drinkers), and calibrated intakes of energy, fruit and vegetables combined, sugars (as % energy), fibre from cereals, and each other food (each continuous), and stratified by sex and EPIC centre.

[#] Tests of trend were based on the calibrated intake of each food in each category.

[&] Tests of heterogeneity of trend by prior disease status were obtained assuming independence of risk between the categories.

Supplemental Table 3 Mutually-adjusted hazard ratios* (95% confidence intervals) for first non-fatal MI or fatal IHD per increment in calibrated intake of selected animal foods, subdivided by smoking status in 400112 participants with known smoking status

Food	Increment (g/day)	Never smoker No. of cases	Never smoker HR (95% CI)	Never smoker P for trend#	Former smoker No. of cases	Former smoker HR (95% CI)	Former smoker P for trend#	Current smoker No. of cases	Current smoker HR (95% CI)	Current smoker P for trend#	P for hetero-geneity&
Red and processed meat	100	1997	1.19 (0.93-1.52)	0.17	2224	1.20 (0.98-1.47)	0.078	2906	1.17 (0.99-1.39)	0.063	0.99
Poultry meat	20	1997	1.05 (0.94-1.16)	0.41	2224	0.99 (0.91-1.07)	0.82	2906	0.93 (0.85-1.02)	0.12	0.26
White fish	15	1997	0.97 (0.90-1.04)	0.41	2224	1.02 (0.95-1.08)	0.65	2906	1.02 (0.97-1.08)	0.44	0.51
Fatty fish	15	1997	0.96 (0.88-1.05)	0.35	2224	1.01 (0.94-1.09)	0.78	2906	0.93 (0.87-1.00)	0.046	0.30
Milk	200	1997	1.06 (0.99-1.14)	0.082	2224	1.02 (0.96-1.09)	0.46	2906	1.01 (0.97-1.06)	0.57	0.51
Yogurt	100	1997	1.03 (0.94-1.13)	0.51	2224	0.95 (0.87-1.04)	0.25	2906	0.86 (0.79-0.93)	0.0004	0.016
Cheese	30	1997	0.86 (0.75-0.99)	0.038	2224	0.99 (0.88-1.12)	0.91	2906	0.89 (0.81-0.98)	0.014	0.24
Eggs	20	1997	1.00 (0.88-1.13)	0.96	2224	0.95 (0.85-1.05)	0.31	2906	0.90 (0.82-0.98)	0.020	0.40

* Hazard ratios are adjusted for age (continuous), smoking status and number of cigarettes per day (where appropriate), histories of diabetes, hypertension and hyperlipidemia, Cambridge physical activity index, employment status, level of education completed, BMI (all categorical, with 'unknown' categories added), current alcohol consumption (non-drinkers and sex-specific fifths of intake among drinkers), and calibrated intakes of energy, fruit and vegetables combined, sugars (as % energy), fibre from cereals, and each other food (each continuous), and stratified by sex and EPIC centre.

# Tests of trend were based on the calibrated intake of each food in each category.

& Tests of heterogeneity of trend by smoking status were obtained assuming independence of risk between the categories.

Supplemental Table 4 Mutually-adjusted hazard ratios* (95% confidence intervals) for first non-fatal MI or fatal IHD per increment in calibrated intake of selected animal foods, subdivided by age at recruitment in 409885 participants

Food	Increment (g/day)	Age <55 years No. of cases	Age <55 years HR (95% CI)	Age <55 years P for trend[#]	Age 55-64 years No. of cases	Age 55-64 years HR (95% CI)	Age 55-64 years P for trend[#]	Age ≥65 years No. of cases	Age ≥65 years HR (95% CI)	Age ≥65 years P for trend[#]	P for hetero-geneity[&]
Red and processed meat	100	2115	1.38 (1.15-1.67)	0.0006	3362	1.15 (0.97-1.37)	0.12	1721	0.91 (0.71-1.18)	0.49	0.036
Poultry meat	20	2115	0.96 (0.88-1.06)	0.47	3362	0.96 (0.89-1.04)	0.36	1721	1.05 (0.96-1.14)	0.27	0.29
White fish	15	2115	0.99 (0.94-1.05)	0.82	3362	1.01 (0.96-1.07)	0.60	1721	1.03 (0.94-1.12)	0.52	0.77
Fatty fish	15	2115	0.99 (0.91-1.06)	0.70	3362	0.95 (0.89-1.01)	0.10	1721	0.97 (0.89-1.06)	0.49	0.73
Milk	200	2115	1.00 (0.95-1.06)	0.98	3362	1.04 (0.99-1.08)	0.12	1721	1.02 (0.93-1.11)	0.71	0.64
Yogurt	100	2115	0.93 (0.85-1.03)	0.18	3362	0.94 (0.87-1.00)	0.058	1721	0.90 (0.80-1.02)	0.097	0.87
Cheese	30	2115	0.88 (0.78-0.98)	0.018	3362	0.97 (0.89-1.06)	0.51	1721	0.81 (0.67-0.99)	0.038	0.16
Eggs	20	2115	0.93 (0.84-1.03)	0.19	3362	0.94 (0.86-1.03)	0.19	1721	0.90 (0.79-1.03)	0.12	0.85

* Hazard ratios are adjusted for age (continuous), smoking status and number of cigarettes per day, histories of diabetes, hypertension and hyperlipidemia, Cambridge physical activity index, employment status, level of education completed, BMI (all categorical, with ‘unknown’ categories added), current alcohol consumption (non-drinkers and sex-specific fifths of intake among drinkers), and calibrated intakes of energy, fruit and vegetables combined, sugars (as % energy), fibre from cereals, and each other food (each continuous), and stratified by sex and EPIC centre.

[#] Tests of trend were based on the calibrated intake of each food in each category.

[&] Tests of heterogeneity of trend by age group were obtained assuming independence of risk between the categories.

Supplemental Table 5 Mutually-adjusted hazard ratios* (95% confidence intervals) for first non-fatal MI or fatal IHD per increment in calibrated intake of selected animal foods, subdivided by sex in 409885 participants

Food	Increment (g/day)	Men			Women			P for heterogeneity[&]
		No. of cases	HR (95% CI)	P for trend[#]	No. of cases	HR (95% CI)	P for trend[#]	
Red and processed meat	100	4608	1.16 (1.02-1.32)	0.023	2590	1.33 (1.04-1.71)	0.026	0.34
Poultry meat	20	4608	0.98 (0.93-1.04)	0.54	2590	1.02 (0.91-1.15)	0.69	0.53
White fish	15	4608	1.00 (0.97-1.04)	0.84	2590	1.02 (0.93-1.12)	0.73	0.81
Fatty fish	15	4608	0.97 (0.93-1.02)	0.25	2590	0.92 (0.84-1.01)	0.076	0.30
Milk	200	4608	1.02 (0.98-1.05)	0.37	2590	1.05 (0.98-1.12)	0.19	0.47
Yogurt	100	4608	0.95 (0.89-1.01)	0.077	2590	0.90 (0.82-0.99)	0.030	0.38
Cheese	30	4608	0.93 (0.87-1.01)	0.074	2590	0.86 (0.75-0.99)	0.039	0.34
Eggs	20	4608	0.90 (0.84-0.97)	0.003	2590	1.06 (0.94-1.20)	0.36	0.026

* Hazard ratios are adjusted for age (continuous), smoking status and number of cigarettes per day, histories of diabetes, hypertension and hyperlipidemia, Cambridge physical activity index, employment status, level of education completed, BMI (all categorical, with 'unknown' categories added), current alcohol consumption (non-drinkers and sex-specific fifths of intake among drinkers), and calibrated intakes of energy, fruit and vegetables combined, sugars (as % energy), fibre from cereals, and each other food (each continuous), and stratified by EPIC centre.
[#] Tests of trend were based on the calibrated intake of each food in each category.
[&] Tests of heterogeneity of trend by gender were obtained assuming independence of risk between men and women.

Supplemental Table 6 Mutually-adjusted hazard ratios* (95% confidence intervals) for first non-fatal MI or fatal IHD per increment in calibrated intake of selected animal foods, subdivided by BMI category in 406264 participants with known BMI

Food	Increment (g/day)	BMI <25 kg/m^2 No. of cases	BMI <25 kg/m^2 HR (95% CI)	BMI <25 kg/m^2 P for trend#	BMI 25-29.9 kg/m^2 No. of cases	BMI 25-29.9 kg/m^2 HR (95% CI)	BMI 25-29.9 kg/m^2 P for trend#	BMI ≥30 kg/m^2 No. of cases	BMI ≥30 kg/m^2 HR (95% CI)	BMI ≥30 kg/m^2 P for trend#	P for hetero-geneity&
Red and processed meat	100	2248	1.20 (0.98-1.47)	0.085	3383	1.16 (0.98-1.37)	0.087	1535	1.12 (0.88-1.43)	0.35	0.92
Poultry meat	20	2248	0.95 (0.86-1.04)	0.28	3383	1.00 (0.93-1.07)	0.98	1535	1.01 (0.90-1.13)	0.89	0.64
White fish	15	2248	1.02 (0.94-1.10)	0.63	3383	1.00 (0.95-1.05)	0.99	1535	1.01 (0.95-1.08)	0.71	0.90
Fatty fish	15	2248	0.97 (0.90-1.06)	0.53	3383	0.98 (0.92-1.04)	0.42	1535	0.91 (0.83-1.00)	0.047	0.45
Milk	200	2248	1.02 (0.96-1.08)	0.54	3383	1.03 (0.98-1.07)	0.28	1535	1.02 (0.95-1.09)	0.65	0.97
Yogurt	100	2248	0.89 (0.81-0.97)	0.009	3383	0.95 (0.88-1.02)	0.15	1535	1.00 (0.89-1.12)	0.99	0.26
Cheese	30	2248	0.88 (0.78-0.99)	0.040	3383	0.88 (0.80-0.97)	0.012	1535	1.05 (0.92-1.20)	0.46	0.083
Eggs	20	2248	0.95 (0.85-1.06)	0.38	3383	0.93 (0.85-1.01)	0.091	1535	0.93 (0.82-1.05)	0.25	0.94

* Hazard ratios are adjusted for age (continuous), smoking status and number of cigarettes per day, histories of diabetes, hypertension and hyperlipidemia, Cambridge physical activity index, employment status, level of education completed, BMI (where appropriate; all categorical, with 'unknown' categories added), current alcohol consumption (non-drinkers and sex-specific fifths of intake among drinkers), and calibrated intakes of energy, fruit and vegetables combined, sugars (as % energy), fibre from cereals, and each other food (each continuous), and stratified by sex and EPIC centre.

# Tests of trend were based on the calibrated intake of each food in each category.

& Tests of heterogeneity of trend by BMI category were obtained assuming independence of risk between the categories.

Supplemental Table 7 Mutually-adjusted hazard ratios* (95% confidence intervals) for first non-fatal MI or fatal IHD per increment in calibrated intake of selected animal foods, subdivided by European region in 409885 participants

Food	Increment (g/day)	Northern Europe No. of cases	Northern Europe HR (95% CI)	P for trend#	Central Europe No. of cases	Central Europe HR (95% CI)	P for trend#	Southern Europe No. of cases	Southern Europe HR (95% CI)	P for trend#	P for hetero-geneity&
Red and processed meat	100	3763	1.21 (1.01-1.43)	0.034	1995	1.06 (0.88-1.28)	0.55	1440	1.44 (1.09-1.89)	0.010	0.20
Poultry meat	20	3763	0.98 (0.88-1.10)	0.72	1995	0.97 (0.91-1.04)	0.47	1440	1.06 (0.95-1.18)	0.30	0.43
White fish	15	3763	1.03 (0.97-1.09)	0.38	1995	0.98 (0.85-1.12)	0.76	1440	1.00 (0.95-1.05)	0.95	0.73
Fatty fish	15	3763	0.94 (0.88-1.00)	0.038	1995	0.97 (0.88-1.07)	0.58	1440	1.02 (0.94-1.10)	0.67	0.25
Milk	200	3763	1.02 (0.99-1.06)	0.23	1995	0.97 (0.90-1.05)	0.45	1440	1.11 (1.00-1.23)	0.043	0.12
Yogurt	100	3763	0.93 (0.88-0.99)	0.020	1995	0.88 (0.77-1.02)	0.099	1440	0.97 (0.81-1.17)	0.78	0.70
Cheese	30	3763	0.92 (0.85-1.00)	0.062	1995	0.85 (0.72-1.00)	0.047	1440	0.93 (0.79-1.09)	0.37	0.61
Eggs	20	3763	0.90 (0.82-0.98)	0.013	1995	0.97 (0.87-1.08)	0.60	1440	0.99 (0.88-1.13)	0.91	0.32

* Hazard ratios are adjusted for age (continuous), smoking status and number of cigarettes per day, histories of diabetes, hypertension and hyperlipidemia, Cambridge physical activity index, employment status, level of education completed, BMI (where appropriate; all categorical, with 'unknown' categories added), current alcohol consumption (non-drinkers and sex-specific fifths of intake among drinkers), and calibrated intakes of energy, fruit and vegetables combined, sugars (as % energy), fibre from cereals, and each other food (each continuous), and stratified by sex and EPIC centre.

# Tests of trend were based on the calibrated intake of each food in each region.

& Tests of heterogeneity of trend by European region (Northern: Denmark, Norway, Sweden [Malmö]; Central: France excepting Provence and SW France, Netherlands, UK; Southern: Greece, Italy, Spain, Provence, SW France) were obtained assuming independence of risk between the regions.

Supplemental Table 8. Hazard ratios* (95% confidence intervals) for first non-fatal MI or fatal IHD per increment in calibrated intake of selected animal foods, with mutual adjustment for the calibrated intakes of each other food, subdivided by extent of CHD event validation

Food	Increment (g/day)	Partial validation			Complete validation			P for heterogeneity[&]
		No. of cases	HR (95% CI)	P for trend[#]	No. of cases	HR (95% CI)	P for trend[#]	
Red and processed meat	100	3798	1.06 (0.91-1.22)	0.48	3400	1.43 (1.19-1.73)	0.0001	0.011
Poultry meat	20	3798	0.97 (0.91-1.03)	0.37	3400	1.04 (0.94-1.14)	0.44	0.26
White fish	15	3798	1.02 (0.97-1.08)	0.44	3400	1.00 (0.96-1.05)	0.86	0.62
Fatty fish	15	3798	0.95 (0.89-1.01)	0.089	3400	0.98 (0.92-1.04)	0.54	0.46
Milk	200	3798	0.97 (0.92-1.02)	0.29	3400	1.06 (1.01-1.10)	0.008	0.014
Yogurt	100	3798	0.94 (0.87-1.01)	0.097	3400	0.92 (0.86-0.99)	0.023	0.66
Cheese	30	3798	0.87 (0.78-0.97)	0.016	3400	0.94 (0.87-1.02)	0.16	0.26
Eggs	20	3798	0.94 (0.87-1.03)	0.17	3400	0.93 (0.85-1.01)	0.088	0.79

* Hazard ratios are adjusted for age (continuous), smoking status and number of cigarettes per day, histories of diabetes, hypertension and hyperlipidaemia, Cambridge physical activity index, employment status, level of education completed, BMI (where appropriate; all categorical, with 'unknown' categories added), current alcohol consumption (non-drinkers and sex-specific fifths of intake among drinkers), and calibrated intakes of energy, fruit and vegetables combined, sugars (as % energy) and fibre from cereals (each continuous), and each other food, as appropriate (each continuous), and stratified by sex and EPIC centre. Results are based on 7198 cases among 409,885 participants.
[#] Tests of trend were based on the calibrated intake of each food in each region.
[&] Tests of heterogeneity of trend by extent of CHD event validation (partial: France, Netherlands, Sweden, UK; complete: Denmark, Greece, Italy, Norway, Spain) were obtained assuming independence of risk between the categories.

Supplemental Table 9 Mean* non-HDL cholesterol (mmol/l; 95% confidence intervals) by overall fifths of observed intake of selected animal foods in up to 16421 participants in the sub-cohort

Food	No. of observations	Fifth of intake					P for trend[#]
		1	2	3	4	5	
Red and processed meat	16421	4.37 (4.33-4.41)	4.41 (4.37-4.45)	4.43 (4.39-4.47)	4.52 (4.48-4.55)	4.56 (4.51-4.60)	<0.0001
Red meat	16421	4.39 (4.35-4.43)	4.39 (4.35-4.43)	4.45 (4.41-4.49)	4.51 (4.47-4.54)	4.55 (4.51-4.59)	<0.0001
Processed meat	16421	4.38 (4.34-4.43)	4.43 (4.39-4.47)	4.48 (4.44-4.52)	4.47 (4.43-4.51)	4.52 (4.48-4.56)	0.0001
Poultry meat	16421	4.41 (4.36-4.45)	4.44 (4.40-4.48)	4.45 (4.42-4.49)	4.47 (4.43-4.51)	4.52 (4.48-4.56)	0.0002
White fish	14280	4.43 (4.38-4.48)	4.40 (4.35-4.45)	4.45 (4.41-4.50)	4.47 (4.42-4.51)	4.49 (4.44-4.54)	0.025
Fatty fish	15298	4.40 (4.35-4.44)	4.44 (4.40-4.48)	4.46 (4.42-4.50)	4.50 (4.46-4.54)	4.49 (4.45-4.53)	0.006
Milk	16421	4.48 (4.44-4.52)	4.51 (4.47-4.55)	4.47 (4.43-4.51)	4.40 (4.36-4.44)	4.43 (4.39-4.47)	0.004
Yogurt	16421	4.48 (4.44-4.52)	4.51 (4.46-4.56)	4.48 (4.44-4.52)	4.44 (4.00-4.47)	4.38 (4.34-4.42)	<0.0001
Cheese	16421	4.52 (4.48-4.56)	4.50 (4.46-4.54)	4.42 (4.38-4.46)	4.42 (4.38-4.46)	4.42 (4.38-4.46)	0.0006
Eggs	16421	4.49 (4.45-4.54)	4.48 (4.44-4.52)	4.45 (4.41-4.49)	4.46 (4.42-4.50)	4.41 (4.37-4.45)	0.007

* Means are adjusted for age (continuous), sex and EPIC centre.
[#] based on median food intake in each category.

Supplemental Table 10 Mean* systolic blood pressure (mm Hg; 95% confidence intervals) by overall fifths of observed intake of selected animal foods in up to 13294 participants in the sub-cohort

Food	No. of observations	Fifth of intake					P for trend#
		1	2	3	4	5	
Red and processed meat	13294	130.3 (129.6-131.0)	131.9 (131.2-132.5)	132.5 (131.9-133.2)	132.2 (131.6-132.9)	133.6 (132.9-134.3)	<0.0001
Red meat	13294	130.5 (129.8-131.2)	131.9 (131.2-132.6)	132.5 (131.9-133.2)	132.7 (132.1-133.4)	132.7 (132.0-133.4)	0.0005
Processed meat	13294	130.0 (129.2-130.8)	132.1 (131.5-132.8)	132.3 (131.6-133.0)	132.6 (131.9-133.3)	133.7 (132.9-134.5)	<0.0001
Poultry meat	13294	131.7 (131.0-132.4)	131.9 (131.3-132.6)	132.0 (131.4-132.6)	132.3 (131.6-133.0)	132.7 (131.9-133.5)	0.048
White fish	11617	132.1 (131.2-132.9)	131.2 (130.3-132.1)	131.9 (131.1-132.7)	132.1 (131.3-132.9)	132.5 (131.5-133.4)	0.21
Fatty fish	12220	132.0 (131.2-132.7)	132.2 (131.5-132.9)	132.0 (131.3-132.7)	131.9 (131.2-132.7)	131.8 (131.0-132.6)	0.61
Milk	13294	132.1 (131.4-132.8)	132.1 (131.4-132.8)	132.2 (131.5-132.9)	131.7 (131.0-132.4)	132.3 (131.6-133.0)	0.78
Yogurt	13294	133.0 (132.2-133.7)	132.1 (131.3-132.8)	132.1 (131.4-132.7)	132.2 (131.5-132.9)	131.2 (130.6-131.9)	0.003
Cheese	13294	132.2 (131.4-133.0)	132.7 (132.0-133.3)	132.0 (131.3-132.6)	131.8 (131.2-132.5)	131.8 (131.2-132.5)	0.24
Eggs	13294	132.0 (131.3-132.7)	131.6 (131.0-132.3)	132.0 (131.4-132.7)	132.0 (131.3-132.7)	133.0 (132.3-133.8)	0.015

* Means are adjusted for age (continuous), sex and EPIC centre.
# based on median food intake in each category.
